# TALEN‐mediated gene editing of the *thrombospondin‐1* locus in axolotl

**DOI:** 10.1002/reg2.29

**Published:** 2015-04-08

**Authors:** Tzu‐Hsing Kuo, Johanna E. Kowalko, Tia DiTommaso, Mandi Nyambi, Daniel T. Montoro, Jeffrey J. Essner, Jessica L. Whited

**Affiliations:** ^1^ Brigham Regenerative Medicine Center and Department of Orthopedic SurgeryBrigham and Women's HospitalHarvard Medical SchoolHarvard Stem Cell InstituteCambridgeMassachusetts02139USA; ^2^ Department of GeneticsDevelopment and Cell BiologyIowa State UniversityAmesIowa50011USA

**Keywords:** Axolotl, gene targeting, genome editing, limb, regeneration, TALEN, *thrombospondin‐1*

## Abstract

Loss‐of‐function genetics provides strong evidence for a gene's function in a wild‐type context. In many model systems, this approach has been invaluable for discovering the function of genes in diverse biological processes. Axolotls are urodele amphibians (salamanders) with astonishing regenerative abilities, capable of regenerating entire limbs, portions of the tail (including spinal cord), heart, and brain into adulthood. With their relatively short generation time among salamanders, they offer an outstanding opportunity to interrogate natural mechanisms for appendage and organ regeneration provided that the tools are developed to address these long‐standing questions. Here we demonstrate targeted modification of the *thrombospondin‐1* (*tsp‐1*) locus using transcription‐activator‐like effector nucleases (TALENs) and identify a role of *tsp‐1* in recruitment of myeloid cells during limb regeneration. We find that while *tsp‐1*‐edited mosaic animals still regenerate limbs, they exhibit a reduced subepidermal collagen layer in limbs and an increased number of myeloid cells within blastemas. This work presents a protocol for generating and genotyping mosaic axolotls with TALEN‐mediated gene edits.

## Significance

An axolotl can regenerate many parts of its body including entire limbs and portions of the heart, brain, spinal cord (Seifert & Voss 2013), making it a very attractive model to study regeneration. In recent years, several modern techniques have been applied to axolotls, allowing for the functional study of specific genes. For example, plasmids containing genes of interest can be delivered to local tissues by virus infection (Whited et al. 2013) or electroporation (Echeverri & Tanaka 2003) and foreign DNA can also be injected into single‐cell‐stage embryos to generate transgenic animals and inducible transgenic animals (Sobkow et al. 2006; Whited et al. 2012; Khattak et al. 2013). However, all these methods involve introducing exogenous DNA, and it will be imperative to also be able to decrease or eliminate gene function to fully understand regeneration. Morpholinos, antisense oligonucleotides that block the access of mRNA and decrease protein expression, have enabled local and transient knockdown of specific gene activities in salamanders (Schnapp & Tanaka 2005; Tsonis et al. 2011; Zhu et al. 2012). While useful, this method is not permanent, and many genes likely to be important may not be sufficiently knocked down to impair regeneration. Furthermore, morpholinos can sometimes produce off‐target effects, and the method is labor‐intensive as the treatment needs to be administered with each experiment. The development of an alternative method to examine loss of function is of particular interest in this field, and one exciting possibility is to develop methods for targeting and editing endogenous axolotl genomic loci.

Transcription‐activator‐like effector nucleases (TALENs) have been successfully applied to several animal models such as *Caenorhabditis elegans*, *Drosophila*, zebrafish, *Xenopus*, mouse and rat (Huang et al. 2011; Sander et al. 2011; Lei et al. 2012; Liu et al. 2012; Ferguson et al. 2013; Sung et al. 2013; Miki et al. 2014; Sugi et al. 2014) and also two species of salamanders, Iberian ribbed newts (Hayashi et al. 2014) and axolotls (Fei et al. 2014), for site‐specific gene targeting and editing. TALENs are engineered DNA nucleases that contain two effective components: a customized DNA binding domain derived from transcription‐activator‐like (TAL) effectors and a DNA nuclease derived from FokI endonuclease that mediates double‐strand breaks. TAL effectors are originally from plant pathogenic bacteria *Xanthomonas* (Bogdanove et al. 2010), and their recognition of DNA is mediated by repeat‐variable di‐residue (RVD) (Boch et al. 2009; Moscou & Bogdanove 2009), by which one RVD recognizes one nucleotide. Double‐strand breaks are generated when DNA is targeted by TALENs and lead to two highly conserved DNA repair processes: non‐homologous end joining, which is error‐prone and often results in insertions or deletions (indels) that can result in frame shifts or a premature stop codon, or homologous recombination, which leads to high fidelity DNA repair but occurs at a lower rate. In the presence of an exogenously introduced homologous sequence flanking the cleavage site, the homologous recombination process can be used for precise gene modification.

We have sought to implement TALEN‐mediated gene editing to create loss‐of‐function alleles of axolotl *thrombospondin‐1* and thereby enable elucidation of its role in regeneration. Thrombospondin‐1 (TSP‐1) is an extracellular matrix protein that belongs to the highly conserved thrombospondin family (reviewed in Adams & Lawler 2011). TSP‐1 has numerous and diverse functions in mammals; for example, it is involved in platelet aggregation, inflammation, wound healing, angiogenesis, tumor progression, and cardiovascular diseases (reviewed in Esemuede et al. 2004; Lopez‐Dee et al. 2011). Recently, TSP‐1 has been shown to be involved in regeneration in other organisms. TSP‐1 expressed from endothelial cells supports the differentiation of murine lung epithelial stem cells (Lee et al. 2014) and some reports indicate that TSP‐1 inhibits liver cell proliferation during liver regeneration (Hayashi et al. 2012; Starlinger et al. 2013). We have previously shown that the expression of *tsp‐1* is induced during axolotl limb regeneration (Whited et al. 2011). In this report, we successfully edited the axolotl *tsp‐1* locus using the Golden Gate method (Cermak et al. 2011) for assembly of TALENs and subsequent injection into single‐celled embryos. We found that mosaic depletion of TSP‐1 results in decreased collagen thickness beneath the epidermis in the limb as well as increased numbers of myeloid cells within the blastema, the collection of limb progenitor cells at the tip of the stump.

## Results

### Strategy for designing TALENs targeting axolotl *thrombospondin‐1*

To design the DNA recognition sequences for TALENs, we first identified a relatively long exon within the axolotl *tsp‐1* gene amenable to polymerase chain reaction (PCR) amplification and containing potential TALEN target sites. Because the genome of axolotl has not been sequenced, we aligned axolotl *tsp‐1* cDNA (Genbank accession number HQ380179; Whited et al. 2011) to the *Xenopus laevis tsp‐1* open reading frame and identified possible exon junctions. We confirmed the junctions by sequencing PCR products amplified from genomic DNA (data not shown).

**Figure 1:**
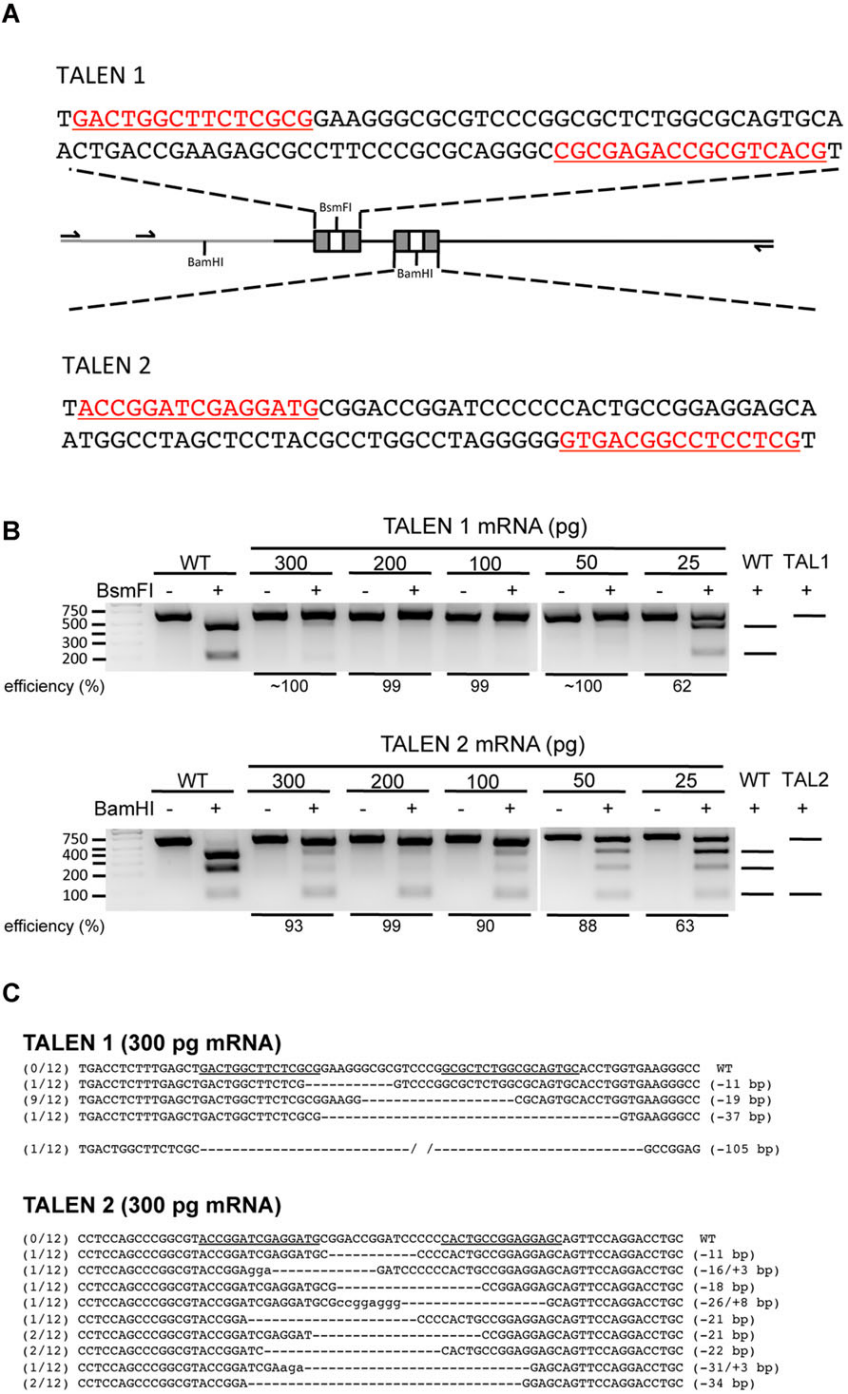
Editing axolotl *tsp‐1* locus by TALENs. (A) Schematic of the exonic region in axolotl *tsp‐1* locus and design of TALENs. The exonic region of *tsp‐1* locus is indicated by the black line and the intronic region is indicated by the gray line. The binding sequences of the TALEN pairs used in this study are underlined and highlighted in red or marked as gray boxes. Primers used for PCR reactions are indicated by arrows, and the recognition sites of restriction enzymes used for determining efficiency of editing are noted. (B) Cleavage of PCR products by restriction enzymes indicates that the *tsp‐1* locus has been edited. Genomic DNA from one embryo of WT (wild‐type; non‐injected) or TALEN mRNA injection is used for each lane. For each sample, an equal amount of PCR product, not incubated with the restriction enzyme, was loaded as an undigested control. The predicted patterns of DNA fragments after restriction enzyme digestion are shown on the right. First lane from the left is the DNA ladder. PCR amplicons from TALEN1 and TALEN2 mRNA‐injected embryos were cleaved by *Bsm*FI or *Bam*HI, respectively. (C) Sequencing results of the PCR amplicons from *tsp‐1* locus of embryos injected with 300 pg TALEN mRNA, which shows various indel mutations in the *tsp‐1*‐TALEN target site. The numbers listed at the beginning of each sequence indicate the frequency of that sequence being detected among all sequenced amplicons for each embryo. Sequences recognized by TALEN pairs are underlined in WT sequences. Lower‐case letters indicate insertions.

Next, we designed two sets of TALEN pairs targeting sites close to the 5′ end of this predicted exonic region (Fig. 1A). The TALENs work in pairs. Each monomer contains an array of RVDs to bind DNA sequences on the opposite strand. The nuclease activity is mediated by dimerization of FokI, which results from proper orientation of each TALEN monomer and proper length of the spacer region (DNA sequence between two DNA binding sites). We designed each TALEN pair to have 15–17 RVDs that recognize and bind DNA, and with a spacer of 15 bp between two TALEN monomers. The plasmids containing proper RVDs to recognize the *tsp‐1* locus were then assembled by the Golden Gate method described previously (Cermak et al. 2011). The TALEN mRNAs were transcribed and purified *in vitro*.

### Embryos targeted by TALENs showed indel mutations

To determine the optimal amount of mRNA for TALENs, single‐cell‐staged embryos were first injected with 300, 200, 100, or 50 pg of TALEN mRNAs, or left uninjected. Most embryos survived at 2 days post‐injection (Table 1). Two apparently healthy, well‐developed embryos of each group were harvested for genomic DNA isolation, while the remaining embryos were allowed to continue to develop. We used restriction enzyme digestion to determine if the target site within the *tsp‐1* locus was edited. The wild‐type sequence includes a specific restriction enzyme recognition site that is cleaved in the PCR product from unedited alleles, resulting in smaller, digested bands on a gel compared to undigested PCR product. Edited alleles may delete the restriction enzyme recognition site, resulting in the presence of uncleaved PCR products following incubation with the enzyme. Genome editing was observed in all harvested embryos injected with various amounts of RNA ranging from 50 to 300 pg per embryo (Fig. 1B). Higher efficiency was observed in embryos injected with more mRNA (Fig. 1B). However, higher mortality was also observed in those conditions (Table 1). Still, we observed editing at 25 pg, with approximately 62% and 63% efficiency in embryo preparations from TAL1 and TAL2 injections, respectively, as determined by restriction enzyme digestion (Fig. 1B).

**Table 1:** Survival rate of embryos injected with various concentrations of TALEN mRNA or left uninjected. For each condition, up to 10 individual embryos were injected initially. Embryos without successful fertilization were excluded from the experiment. At two days post‐injection, two embryos from each group were harvested for DNA isolation and the subsequent survival rate was determined according to the adjusted total animal numbers. Survival was determined by visual inspection

		Survival rate					
	Total mRNA injected (pg)	1 day	2 days^a^	4 days	6 days	15 days	18 weeks
WT	N.A.	86% (6/7)	80% (4/5)	80% (4/5)	80% (4/5)	80% (4/5)	80% (4/5)
TALEN1	300	88% (7/8)	83% (5/6)	0% (0/6)	0% (0/6)	0% (0/6)	0% (0/6)
	200	90% (9/10)	88% (7/8)	13% (1/8)	13% (1/8)	13% (1/8)	13% (1/8)
	100	100% (9/9)	100% (7/7)	43% (3/7)	29% (2/7)	14% (1/7)	0% (0/7)
	50	100% (8/8)	100% (6/6)	83% (5/6)	83% (5/6)	67% (4/6)	33% (2/6)
TALEN2	300	100% (8/8)	33% (2/6)	0% (0/6)	0% (0/6)	0% (0/6)	0% (0/6)
	200	88% (7/8)	67% (4/6)	0% (0/6)	0% (0/6)	0% (0/6)	0% (0/6)
	100	100% (8/8)	100% (6/6)	50% (3/6)	50% (3/6)	0% (0/6)	0% (0/6)
	50	100% (8/8)	83% (5/6)	67% (4/6)	50% (3/6)	50% (3/6)	50% (3/6)

a. Two embryos from each group were harvested for DNA isolation at 2 days post‐injection. Remaining animals (N‐2) were subsequently used to determine survival rate. N = initial number of embryos injected.

To further confirm that the *tsp‐1* locus was edited by TALENs, the PCR amplicon was cloned into a TA cloning vector and sequenced. Indeed, embryos showing evidence of editing by restriction enzyme digestion also have insertion or deletion mutations, which were revealed by sequencing (Figs. 1C and S2). The sequencing results also confirmed the dosage‐dependent effect of TALEN mRNA on genome‐editing efficiency.

**Figure 2:**
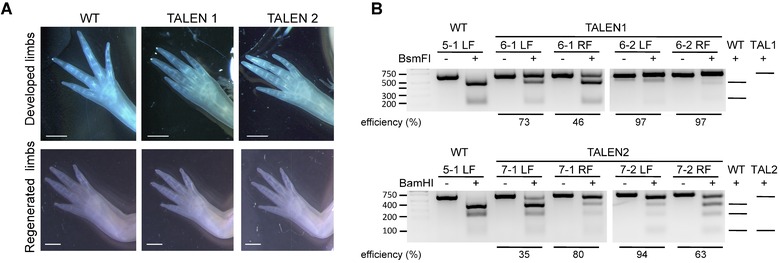
TALEN‐targeted animals show edited *tsp‐1* locus in the limbs and normal limb morphology. (A) Morphology of developed limbs and regenerated limbs 6 weeks post‐amputation from wild‐type and TALEN‐targeted animals. Scale bar indicates 1 mm. (B) PCR amplicon of the *tsp‐1* locus from limbs from wild‐type and TALEN‐injected juvenile animals digested by restriction enzyme indicated edited *tsp‐1* locus. Genomic DNA from one forelimb of WT (wild‐type; non‐injected) or TALEN mRNA‐injected animals was used in each lane. For each sample, an equal amount of PCR product, not incubated with the restriction enzyme, was loaded as an undigested control. The predicted patterns of DNA fragments after restriction enzyme digestion are illustrated on the right. The first lane from the left is the DNA ladder. PCR amplicons from TALEN1‐ and TALEN2‐mRNA‐injected embryos were cleaved by *Bsm*FI or *Bam*HI, respectively.

### TALEN‐targeted cells persist during development and are found in juvenile limbs

Next, we examined the effect of embryonic TALEN injections in juvenile axolotl limbs. Similar to the phenotype observed in *tsp‐1* knockout mice (Lawler et al. 1998), no obvious developmental defects were observed in the limbs of *tsp‐1* TALEN‐targeted juvenile axolotls (Fig. 2A). We amputated all four limbs of each animal to examine the effect of *tsp‐1* gene editing on limb regeneration. Limb tissues near the amputation plane were harvested for DNA extraction. PCR amplicons of the region flanking *tsp‐1* edits were subjected to restriction enzyme digestion, which revealed that limb tissues near the amputation plane were also edited by TALENs with high efficiency (ranging from 35% to 97%, Fig. 2B). We calculated the average editing efficiency as 72 ± 9% (*N* = 6 limbs) for TAL1‐edited limbs and 77 ± 6% (*N* = 16) for TAL2‐edited limbs. PCR amplicons from each of the TALEN‐edited limbs were also cloned and sequenced. We found that limbs showing evidence of editing by restriction enzyme digestion do indeed have insertion or deletion mutations (Fig. S2). However, those limbs regenerated normally compared to non‐TALEN‐targeted control limbs (Fig. 2A, lower panel). In these mosaic animals, even with many cells probably harboring defective coding sequences for TSP‐1, a threshold number of cells producing wild‐type TSP‐1 protein may enable relatively normal regeneration. However, it is also possible that in a complete loss‐of‐function setting, with all cells defective in TSP‐1 production, limbs may regenerate normally and TSP‐1 may be dispensable for regeneration. Distinguishing between these possibilities will require breeding the animals to produce individuals whose genotype is *tsp‐1^−/+^* or possibly *tsp‐1^−/−^* in all cells.

### *Thrombospondin‐1* edited limbs have specific cellular differences compared to wild‐type

**Figure 3:**
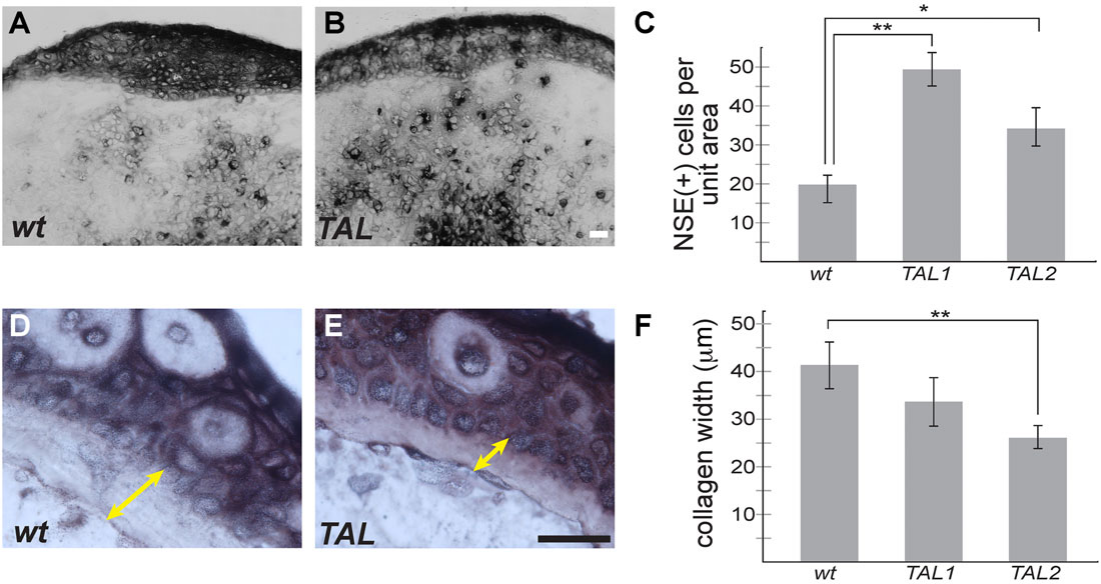
TALEN‐targeted animals show increased macrophage and monocyte infiltration in regenerating limbs and decreased stump collagen deposition. NSE staining was performed to detect monocytes and macrophages in regenerating juvenile limbs at 6 days post‐amputation. (A) Wild‐type sibling control. (B) TALEN‐targeted *tsp‐1* deletion animal. (C) The quantification of NSE positive cells (A, B, black) within the blastema mesenchyme were quantified (*N* = 14, 6, 16 limbs for *wt*, *TAL1* and *TAL2* respectively). (D), (E) Subepidermal collagen thickness was measured (yellow double arrow) in control and TALEN‐targeted stumps, and quantified in (F) (*N* = 14 controls; 6 *TAL1*; 16 *TAL2*). Scale bars in all images are 50 μm; *P < 0.05, **P < 0.01; error bars indicate SEM.

We sought to determine if there was an observable cellular effect of mosaic loss of *tsp‐1* activity. We histologically analyzed regenerated limbs from TALEN‐edited animals and compared them to limbs from wild‐type siblings. It has recently been shown that macrophages are required for axolotl limb regeneration (Godwin et al. 2013). Furthermore, the *tsp1* null mice show increase in several myeloid lineages (Lawler et al. 1998). Therefore, we performed a non‐specific esterase stain (NSE) to identify macrophages and monocytes in the regenerating limbs of *tsp‐1*‐edited animals and their wild‐type siblings (Fig. 3A−C). We found that *tsp‐1*‐edited animals exhibited a statistically significant increase in the number of NSE(+) cells in the central blastema of limbs at 6 days post‐amputation compared to wild‐type. We also noticed that the collagen fibril layer beneath the stump epidermis appeared thinner in the *tsp‐1*‐edited limbs, and this observation was confirmed by measuring the thickness of this tissue layer. *Tsp‐1*‐edited animals exhibited a nearly twofold reduction in the thickness of subepidermal collagen compared to wild‐type (Fig. 3D−F). Future work will be necessary to determine the implication of these differences upon regeneration or development.

### Future directions

Several powerful methods for genome editing have recently been developed, and new work to improve each is ongoing. The CRISPR system has recently been applied to axolotls using wild‐type *Streptococcus pyogenes* Cas9 nuclease (Flowers et al. 2014). The CRISPR system is an attractive editing technique because it is relatively simple, fast, and cheap to design and generate targeting components. While wild‐type Cas9 provides high editing frequency, it can also induce off‐target indel mutations (Fu et al. 2013; Hsu et al. 2013; Pattanayak et al. 2013) due to the tolerance of mismatch in the guide RNA−DNA (gRNA−DNA) interaction. Off‐target effects are especially problematic in species where the genome is unsequenced (and they are therefore less predictable) and the generation time is long (and hence simply “crossing out” the off‐target lesions may be logistically very difficult), such as in axolotl. The specificity of CRISPR can be increased by using paired Cas9 nickase (Mali et al. 2013; Ran et al. 2013; Cho et al. 2014), truncated‐gRNA (Fu et al. 2014), and RNA‐guided FokI nucleases (Tsai et al. 2014). However, while these modifications increase specificity, they also show reduced editing efficiency.

Both the TALEN and CRISPR techniques have been shown to offer high genome‐editing efficiency (e.g., Li 2011; Lei et al. 2012; Ding et al. 2013; Smith et al. 2014; Veres et al. 2014). Degeneracy in RVD‐DNA binding has been shown in TALENs (Bogdanove & Voytas 2011), but little evidence of off‐target and mismatch has been shown. In contrast to CRISPR techniques, TALENs require a slightly longer time to construct the DNA binding motif. However, the selection of a DNA binding target is less constrained in the TALEN methodology compared to CRISPR. CRISPR gRNA must be designed to immediately precede an NGG (Protospacer Adjacent Motif, PAM) sequence. The high specificity CRISPR platforms require two gRNAs (which means two PAM sequences) to be located within a designated distance (Mali et al. 2013; Ran et al. 2013). In contrast, TALENs can target almost all sites in the genome. The ability to target nearly any sequence may be extremely important for creating engineered mutations.

As both the TALEN and CRISPR technologies are improved upon, developing both technologies is a wise investment for the axolotl community. Crucially, for many if not most genes of interest, determining the effect of gene loss on regeneration will require breeding the edited individuals to homozygosity. For some genes, these technologies may also require layering additional approaches, such as knocking‐in sequence to the locus at the time of editing, for example to enable conditional excision of genes which may be embryonic lethal in the homozygous state. In summary, this report demonstrates another successful application of TALENs and supplements a rising powerful arsenal of tools for studying the remarkable regenerative abilities of axolotls. This report also identifies a role for *tsp‐1* in controlling subepidermal collagen thickness in the limb as well as the amount of myeloid cells localized within the blastema in regenerating limbs. Future studies may determine the mechanisms whereby *tsp‐1* influences these traits and whether they may be involved in tissue homeostasis or regeneration. These future studies will be enabled by the breeding of the mosaic animals to create stable genetic lines of axolotls harboring variant alleles of *tsp‐1*.

## Supporting information

Additional Supporting Information may be found in the online version of this article at the publisher's website:

**Figure S1**. Embryo survival rates.
**Figure S2**. Editing axolotl *tsp‐1* locus by TALENs.
Supplementary Materials and Methods
