# Supplementary material for: TALEN‐mediated gene editing of the thrombospondin‐1 locus in axolotl
Source: Regeneration (Oxf). 2015 Apr 8;2(1):37–43. doi: 10.1002/reg2.29 (PMC4895330; doi:10.1002/reg2.29)

**SUPPLEMENTARY MATERIALS AND METHODS**

**Animal housing**

Axolotls (*wild-type* or *white* mutants, which facilitates microscopic studies) obtained from the Ambystoma Genetic Stock Center (University of Kentucky, Lexington, KY) were bred and raised in our laboratory. Animals are housed in axolotl water (31 mM NaCl, 0.98 mM MgSO_4_, 223 mM CaCl_2_ and 258 mM KCl in deionized water; pH 6.75-7.25 adjusted by adding Sodium bicarbonate (NaHCO_3_)) in a room maintained on a 15 hr:9 hr light:dark cycle at 21°C. Larval and juvenile axolotls were housed in axolotl water containing 1X penicillin (100 units/mL) and streptomycin (100 µg/mL).

**Construction of *tsp1*-targeting TALEN, TALEN mRNA preparation and microinjection**

We used Golden Gate method-based [[1](#_ENREF_1)] assembly to construct of *tsp-1*-targeting TALEN plasmids. Briefly, we designed TALENs towards the 5’ end following the start ATG of the axolotl tsp-1 coding sequence using the TAL Effector Nucleotide Targeter 2.0 (<https://tale-nt.cac.cornell.edu/node/add/talen>) – see citations on website. Repeat arrays for each TALEN half were specified as 15-17 RVDs and a spacer length of 15 base pairs was used. Two TALENs were designed to this locus. For the first TALEN pair, the recognition sequences were: Left TALEN 5’-GACTGGCTTCTCGCG-3’, right TALEN 5’-GCACTGCGCCAGAGCGC-3’, and spacer 5’-GAAGGGCGCGTCCCG-3’. For the second TALEN pair, the recognition sequences were: Left TALEN 5’-ACCGGATCGAGGATG-3’, right TALEN 5’-GCTCCTCCGGCAGTG-3’ and spacer 5’-CGGACCGGATCCCCC-3’. TALENs were constructed as described [25, 26] and cloned into the pT3TS-GoldyTALEN backbone and digested with SacI [26].

mRNAs expressing each *tsp-1*-targeting TALEN were transcribed using the Ambion mMessage Machine T3 kit (Life Technologies) following the manufacturer’s instructions and purified using the RNeasy MinElute Cleanup Kit (Qiagen).

The procedure to inject mRNA into axolotl embryos is previously described [[2](#_ENREF_2)]. Briefly, embryos laid within 30 minutes were collected and stored at 4°C. Embryos that were at single-cell stage were de-jellied. 1.5 nL of mixed TALEN mRNAs were injected into each embryos. 300, 200, 100, 50 or 25 pg of total mRNA (left and right TALEN; half of each) were injected for each TALEN.

**Genomic DNA extraction, PCR and restriction enzyme digestion**

Genomic DNA was isolated from embryos (2 or 7 days post-injection) and limb tissue of juvenile axolotls (5-6 cm snout-to-tail) using DNeasy® Blood & Tissue kit (Qiagen) according to manufacturer's protocol.

PCR reactions to amplify the flanked region of *tsp-1* locus were performed with OneTaq DNA polymerase (New England Biolabs) using the following primers: Forward (5’ TAGTGTCTTCTCCTGACTCTCC 3’) and Reverse (5’ CCCTGTTGAAGAGGTTCTGG 3’). PCR products were then purified by Gel Extraction Kit (Qiagen) and subjected to a second of PCR reaction to further amplify the flanked region of *tsp-1* locus. The second PCR primers were: forward primer (5’ TTCTCTTCCCTGCTATCCCTTAG 3’; downstream of first forward primer, see Figure 1A for the locations of primers) and same reverse primer as the first PCR.

2 μg of purified PCR products were digested by BsmFI (for those targeted by TALEN 1 pairs) or BamHI (for those targeted by TALEN 2 pairs). If the *tsp-1* locus was edited by TALENs, the recognition sites of those restriction enzymes might be deleted, resulting in a resistance of enzyme digestion. Briefly, PCR products were incubated with restriction enzyme or buffer alone according to the manufacturer's protocol. Digested DNA fragments were analyzed by 3.5% agarose gel electrophoresis. The relative intensities of cleaved and uncleaved bands were determined by Image J software.

**Sequencing for edited *tsp-1* locus**

Purified PCR products containing the flanked region of *tsp-1* locus were ligated into a pGEM-T easy TA cloning vector (Promega) and transformed into chemically-competent DH5α cells. Depending on the yield of PCR reaction, the ligated PCR products may come from one or two rounds of PCR reaction, as described previously. Plasmid DNA was isolated and sequenced using T7 or SP6 primers.

**α-Naphtyl Acetate Esterase (NSE) staining**

Non-specific esterase (NSE) was detected using Sigma 90A1-1KT, according to manufacturers’ instructions and [[3](#_ENREF_3)]. Briefly, the blastema of forelimbs from each animals were harvested six days post amputation and freshly frozen in OCT. Tissue were sectioned by cryostat (Leica CM1950) in 16 microns. Sections were post-fixed by citrate-acetone-methanol fixative for 10 minutes. The staining time was reduced to 15 minutes to allow for more accurate counting of positive cells. For each sample, results were quantified by two independent researchers, who counted cells from six sections and averaged the results. Results were statistically analyzed by the nonparametric Mann–Whitney U test.

**REFERENCES**

1. Cermak, T., et al., *Efficient design and assembly of custom TALEN and other TAL effector-based constructs for DNA targeting.* Nucleic Acids Res, 2011. **39**(12): p. e82.

2. Khattak, S., T. Richter, and E.M. Tanaka, *Generation of transgenic axolotls (Ambystoma mexicanum).* Cold Spring Harb Protoc, 2009. **2009**(8): p. pdb.prot5264.

3. Godwin, J.W., A.R. Pinto, and N.A. Rosenthal, *Macrophages are required for adult salamander limb regeneration.* Proc Natl Acad Sci U S A, 2013. **110**(23): p. 9415-20.

Supplementary Figure 1


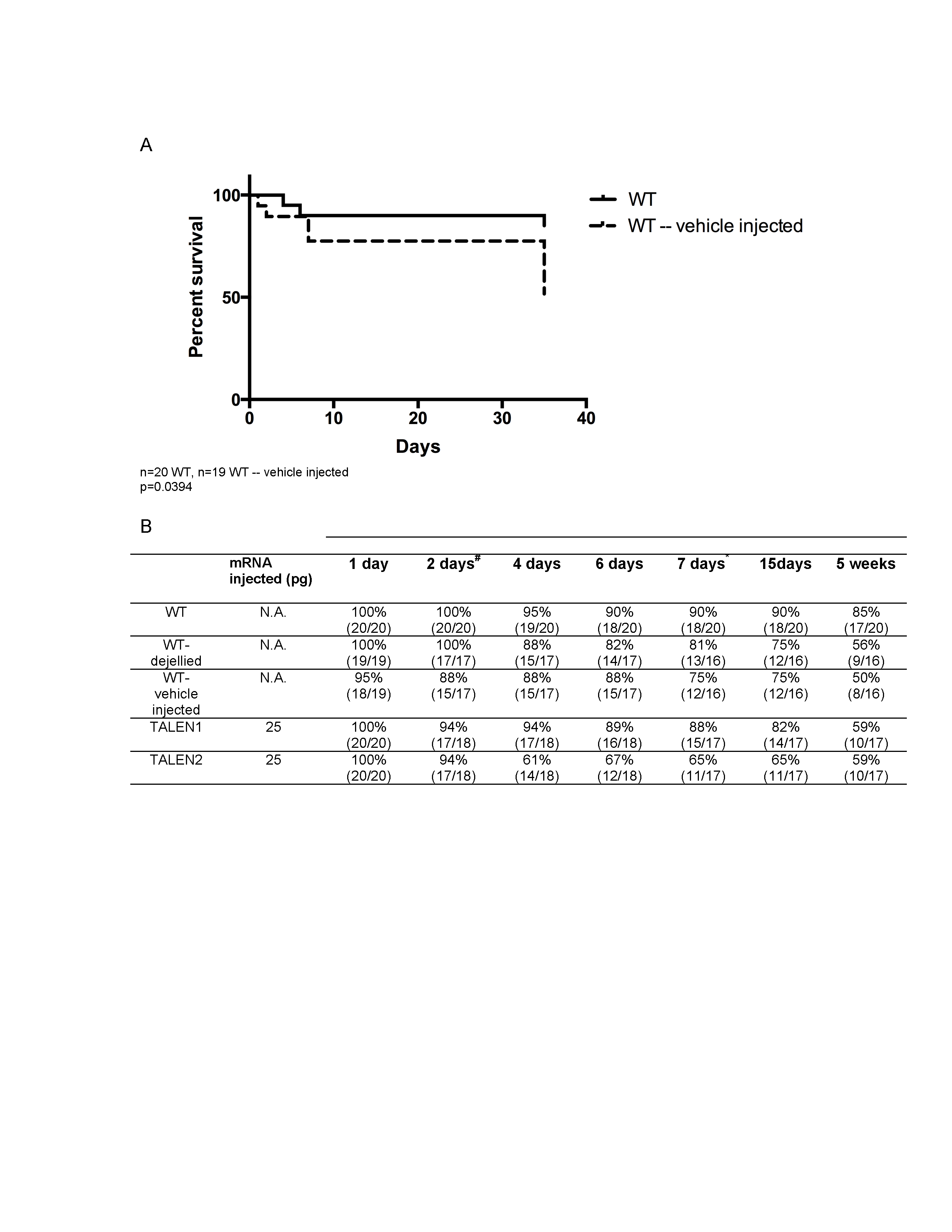


Supplementary Figure 2


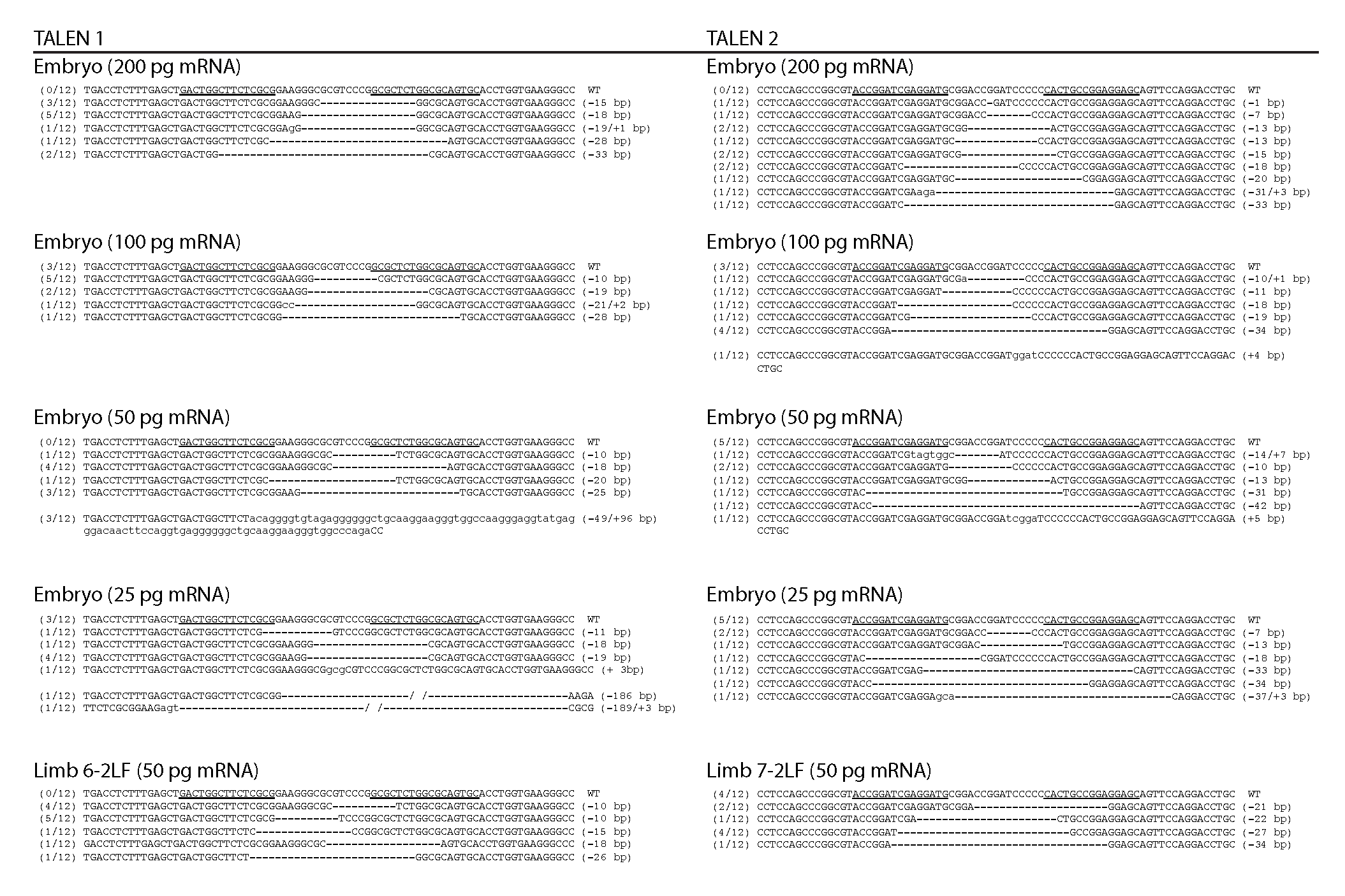

Supplement: Supplementary file 1 — Figure S1. Embryo survival rates. Figure S2. Editing axolotl tsp‐1 locus by TALENs. Supplementary Materials and Methods [file REG2-2-37-s001.docx]
